# Development and investigation of metabolism-associated risk assessment models for patients with viral hepatitis

**DOI:** 10.3389/fcimb.2023.1165647

**Published:** 2023-03-29

**Authors:** Mingjiu Zhao, Yu Lei, Yanyan Zhou, Mingan Sun, Xia Li, Zhiguang Zhou, Jiaqi Huang, Xinyu Li, Bin Zhao

**Affiliations:** ^1^ National Clinical Research Center for Metabolic Diseases, Metabolic Syndrome Research Center, Key Laboratory of Diabetes Immunology, Ministry of Education, and Department of Metabolism and Endocrinology, The Second Xiangya Hospital of Central South University, Changsha, Hunan, China; ^2^ Department of Dermatology, Hunan Key Laboratory of Medical Epigenomics, The Second Xiangya Hospital of Central South University, Changsha, Hunan, China; ^3^ Department of Critical Care Medicine, The Second Xiangya Hospital of Central South University, Changsha, Hunan, China; ^4^ College of Veterinary Medicine, Yangzhou University, Yangzhou, Jiangsu, China; ^5^ Xiangya School of Public Health, Central South University, Changsha, China; ^6^ Furong Laboratory, Central South University, Changsha, China

**Keywords:** viral hepatitis, host-pathogen interactions, metabolism, immune cells, prognosis

## Abstract

Dysregulation of metabolism plays an important role in the onset and progression of multiple pathogenic diseases, including viral hepatitis. However, a model to predict viral hepatitis risk by metabolic pathways is still lacking. Thus, we developed two risk assessment models for viral hepatitis based on metabolic pathways identified through univariate and least absolute shrinkage and selection operator (LASSO) Cox regression analysis. The first model is designed to assess the progression of the disease by evaluating changes in the Child–Pugh class, hepatic decompensation, and the development of hepatocellular carcinoma. The second model is focused on determining the prognosis of the illness, taking into account the patient’s cancer status. Our models were further validated by Kaplan–Meier plots of survival curves. In addition, we investigated the contribution of immune cells in metabolic processes and identified three distinct subsets of immune cells—CD8+ T cells, macrophages, and NK cells—that have significantly affected metabolic pathways. Specifically, our findings suggest that resting or inactive macrophages and NK cells contribute to maintaining metabolic homeostasis, particularly with regard to lipid and α-amino acid metabolism, thereby potentially reducing the risk of viral hepatitis progression. Moreover, maintaining metabolic homeostasis ensures a balance between killer-proliferative and exhausted CD8+ T cells, which helps in mitigating CD8+ T cell-mediated liver damage while preserving energy reserves. In conclusion, our study offers a useful tool for early disease detection in viral hepatitis patients through metabolic pathway analysis and sheds light on the immunological understanding of the disease through the examination of immune cell metabolic disorders.

## 1 Introduction

Viral hepatitis has been a global health problem for a very long time and is still regarded as a serious threat to human health. Hepatitis B and C viruses infect over 300 million people and cause over 1 million deaths annually, leading to significant healthcare and economic burdens for patients and society (Lazarus et al., 2019; The, 2022). Furthermore, patients with viral hepatitis who remain untreated worsen over time and may eventually develop liver cirrhosis and hepatocellular carcinoma (Sherman and Shah, 2018; Lazarus et al., 2019). Despite taking long-term viral suppression treatments, cure rates for hepatitis virus remain low (Manns et al., 2017; Tang et al., 2018), leaving patients to face a long-term risk of disease progression and a shorter life span. In light of these challenges, there is an urgent need for accurate prediction of disease progression and prognosis in patients with viral hepatitis.

X. Chang et al. and X. Zhang et al. developed assessment models of liver injury and cirrhosis of viral hepatitis patients by using selected clinical characteristics (Chang et al., 2021; Zhang et al., 2021). Moreover, researchers have recently built models that take into account factors such as tumor markers, genomes, or transcriptomes for viral hepatitis patients with liver cancer to analyze the risk of carcinogenesis, patient survival, and treatment of liver cancer (Hlady et al., 2019; Xiang et al., 2021; Wang et al., 2022). However, despite advancements made in the field, there are still some crucial elements that influence viral hepatitis progression and prognosis, which have not been incorporated into previous prediction models.

A recent study showed that there is a strong association between chronic hepatitis virus infection and metabolic disorders (Wang et al., 2020). J. Li et al. found that disruptions in lipid and fatty acid metabolism are involved in liver failure progression (Li et al., 2022). Similarly, metabolic reprogramming of cancer cells is well known to have a considerable impact on neoplastic disease initiation and progression. Metabolic changes in other cell types in the tumor microenvironment (TME) can also facilitate tumor development (Martínez-Reyes and Chandel, 2021). Many studies have found that metabolic processes such as bile acid metabolism, amino acid metabolism, and energy metabolism are involved in the occurrence, progression, and patient outcomes of liver cancer, including viral hepatocellular carcinoma (Ma et al., 2018; Dai et al., 2020; Jühling et al., 2021; Liang et al., 2021; Parker et al., 2022). Recent evidence has also suggested that metabolic circuits could be crucial in the treatment of cirrhosis and liver cancer (Zhao et al., 2020; Du et al., 2022). Therefore, we attempted to innovatively develop metabolism prognostic models to offer a clinically novel approach for the risk prediction of viral hepatitis patients.

Additionally, we aimed to explore the connection between metabolism and the progression of viral hepatitis. Metabolism is known to contribute greatly to shaping the body’s immune processes, leading to complex, diverse, and sometimes conflicting results (Patel et al., 2019; Yu et al., 2021). In viral hepatitis patients, there is also a close relationship between metabolism and immunity. For example, M. Canavese et al. found that Trp metabolism participates in both anti-hepatitis C virus (anti-HCV) and anti-tumor immune responses (Canavese et al., 2016). Previous evidence has also shown that disruptions in immunometabolism participate in the process of viral hepatitis exacerbation, hepatocarcinogenesis, and the prognosis of the disease (Wang et al., 2021; Hu et al., 2022; Li et al., 2022). Moreover, metabolic reprogramming of immune cells can play a role in the progression of both hepatitis and liver cancer (Lee et al., 2015; Song et al., 2022). Meanwhile, advances in single-cell RNA sequencing (scRNA-seq) has provided a deeper understanding of immune cell subsets (Papalexi and Satija, 2018). In combination with single-cell analysis, we hope to gain a deeper insight into the role of immunometabolism in disease progression and prognosis.

In this study, we developed metabolism-related models to predict the risk of disease progression and prognosis in viral hepatitis patients. In addition, we investigated its underlying mechanisms by analyzing immune cell infiltration and the intrahepatic single-cell immune landscape. Ultimately, our findings have the potential to facilitate early detection of disease progression in viral hepatitis patients, thereby aiding in the mitigation of disease progression and improving the survival rate of patients.

## 2 Materials and methods

### 2.1 Data preparation

We utilized publicly available datasets to gather mRNA expression profiles and patients’ clinical information from three cohorts. Hepatocellular carcinoma (hcc cohort, Cohort 3) data were obtained from The Cancer Genome Atlas (TCGA), and GSE15654 (cirrhosis cohort, Cohort 2) and GSE84044 (fibrosis cohort, Cohort 1) were obtained from the Gene Expression Omnibus (GEO) database. Gene sets concerning metabolism were retrieved from Molecular Signatures Database (https://www.gsea-msigdb.org/gsea/msigdb/genesets.jsp). Then, single-sample gene set enrichment analysis (ssGSEA) of various metabolic pathways in these cohorts was obtained by using the R package “GSVA”. The databases mentioned above are publicly available. Thus, our study did not require the approval of the local ethics committee.

### 2.2 Risk and prognosis-related candidate selection

Univariate Cox regression was implemented to screen metabolic pathways related to outcomes of patients, such as progression of the Child–Pugh class, hepatic decompensation, hepatocellular carcinoma (HCC) development, and death; p < 0.05 was regarded as statistically significant. Pearson’s correlation analysis was used between ssGSEA and grading of liver biopsy, including the sequential histological staging of fibrosis (Scheuer score “S”) and grading of inflammation (Scheuer score “G”). We screened some metabolic pathways by adjusting p < 0.05 and the absolute value of the correlation coefficient >0.4. Then, we selected risk and prognosis-related overlapping pathways as their respective candidates. Forest plots and heatmap were generated using R software.

### 2.3 Development and validation of the risk and prognosis model

For the metabolic pathways preliminarily screened above, the least absolute shrinkage and selection operator (LASSO) Cox regression analysis and multivariate Cox regression analysis were carried out to identify key pathways and build a prediction model. We calculated the risk score of every patient based on the ssGSEA score of each pathway and its corresponding regression coefficients, score = coef 1 * ssGSEA score of pathway 1 + coef 2 * ssGSEA score of pathway 2 + ··· + coef n * ssGSEA score of pathway n. According to the optimal or median cut point of the risk score in the whole cohort, the patients could be divided into high-risk and low-risk groups. To validate the feasibility of the risk model, the Kaplan–Meier (K-M) survival curve was used between the high-risk and low-risk groups in these datasets separately. According to the progression of the Child–Pugh class-related score of each patient in different Scheuer score subgroups, we made a box plot using GraphPad Prism 9.0 software.

### 2.4 Immune infiltration analysis

With gene expression data in Cohort 1, 22 types of immune cell infiltration of each sample were obtained by CIBERSORTx (https://cibersortx.stanford.edu/index.php). Then, we assessed the relationship between our disease progression risk-related metabolic pathways and immune cell infiltration by using Pearson’s test and adjusting p-value <0.05 and showed results through a heatmap.

### 2.5 Single-cell transcriptomic analysis

To conduct our single-cell analysis, we first utilized data obtained from scRNA-seq results of liver samples sourced from GSE182159, which includes records of 23 individuals (Zhang et al., 2023). To normalize the featured expression measurements for each cell by the total expression, we utilized a global-scaling normalization method called “LogNormalize” and then implemented a linear transformation (scaling) as a standard pre-processing step, calculated variable gene features using the “FindVariableFeatures” function, and conducted principal component analysis on the scaled variable feature data. We then employed the modularity optimization technique “Louvain algorithm” to iteratively cluster cells together, and we used Uniform Manifold Approximation and Projection (UMAP) to visualize similar cells together in low-dimensional space. The gene matrix from all samples was performed by the R package Seurat (Stuart et al., 2019). A multi-dataset integration algorithm “Harmony” was used to correct the batch effect (Korsunsky et al., 2019). Differential gene expression analysis was carried out in Seurat using the “FindMarkers” function with the Wilcoxon test. Differentially expressed genes (DEGs) were ranked by log2-transformed fold change (log2FC) after being filtered with a maximum adjusted p-value of 0.05. Gene set enrichment analysis (GSEA) (Subramanian et al., 2005) was performed to enrich the biological function information of the DEGs. Pathway-specific gene sets were downloaded from the GSEA database (http://www.gsea-msigdb.org). AUCell analysis was performed to evaluate pathway activity based on a given gene set (Aibar et al., 2017). Machine-learning framework (random forest algorithm) was used to quantify the sensitivity and responsiveness of each cell type to biological perturbations in the scRNA-seq dataset, which was calculated by the R package Augur (Skinnider et al., 2021). The area under the receiver operating characteristic curve (AUC) in cross-validation was calculated to characterize the responsiveness. We included metabolic enzyme genes detected by this dataset in machine-learning analysis. The gene list of metabolic enzymes was obtained from the Kyoto Encyclopedia of Genes and Genomes (KEGG) database (https://www.kegg.jp).

### 2.6 Statistical analysis

The statistical analyses were performed using R software (Version 4.1.0, http://www.R-project.org) and the corresponding packages. p-Values less than 0.05 were regarded as statistically significant, and all p-values were bilateral.

## 3 Results

### 3.1 Overview

**Figure 1:**
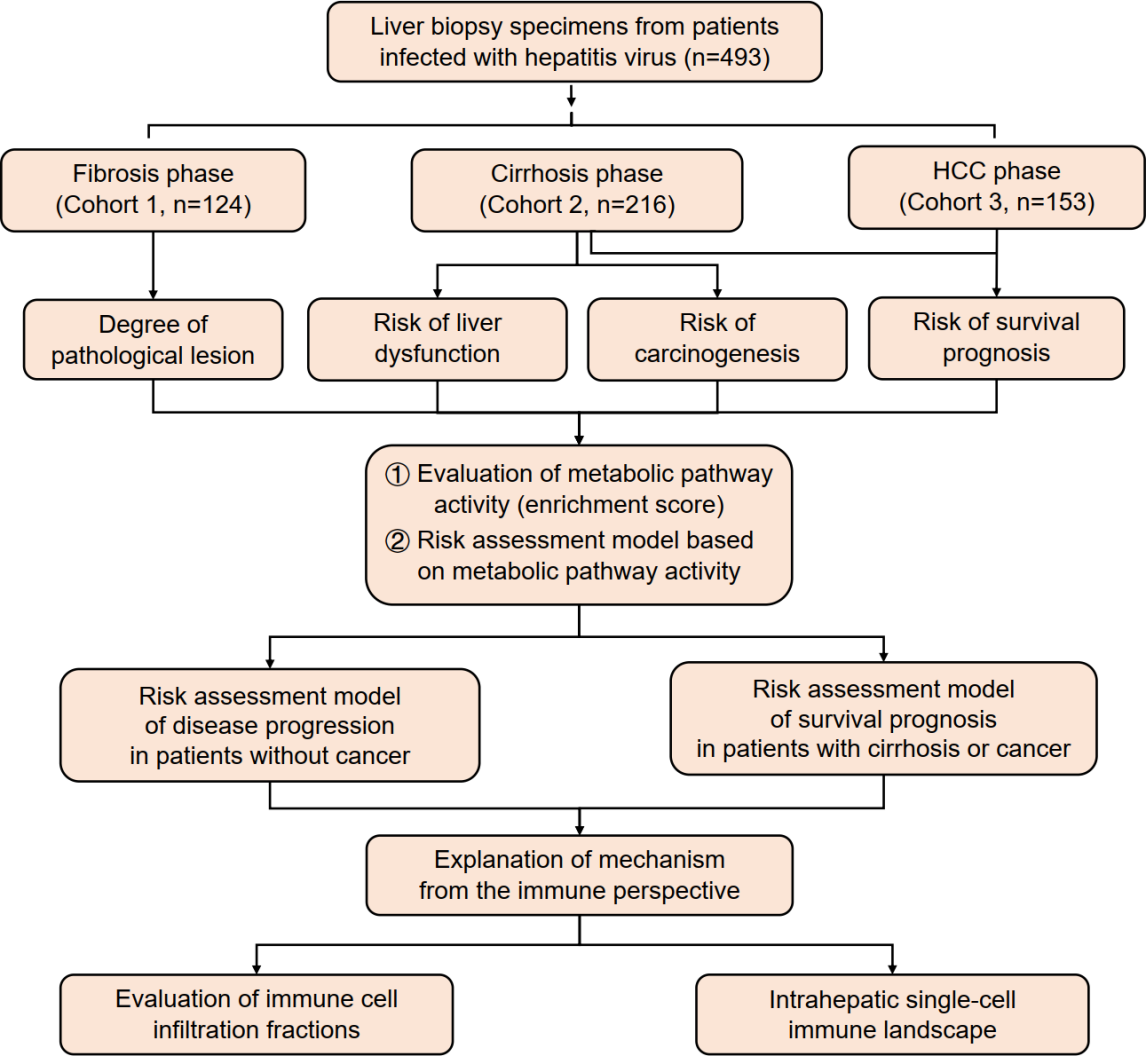
An overview of the study’s design and analysis.

In this study, data were collected from three cohorts of patients infected with hepatitis B virus (HBV)/HCV (n = 493) in different stages of viral hepatitis progression. The activity of metabolic pathways was calculated using ssGSEA score data and analyzed in relation to pathological grades of inflammation and fibrosis, carcinogenesis progression, liver function deterioration, and survival prognosis. A risk assessment model was established based on these findings. Additionally, this study analyzed immune infiltration and created an intrahepatic single-cell immune landscape (**Figure 1**).

### 3.2 Screening disease progression risk-related metabolic pathways in viral hepatitis cohort

First, we examined how different metabolic pathways affected the course of the disease in the cohort of viral hepatitis patients before the cancer stage. The Scheuer scoring system was used to determine the grades of inflammatory activity and degree of fibrosis in Cohort 1 (Scheuer, 1991). We preliminarily screened 220 metabolic pathways associated with both liver inflammation and fibrosis through correlation analysis (|correlation coefficient| > 0.4, p < 0.05) (**Supplementary Table S1**). Furthermore, in Cohort 2, univariate Cox regression analysis was conducted to assess the roles of metabolic pathways in the progression of early-stage (Child–Pugh-A) cirrhosis patients. The main outcome events of disease progression included the progression of the Child–Pugh class (event: child) and hepatic decompensation (event: decomp) and HCC development (event: hcc). The results showed that 96 metabolic pathways (p < 0.05) contributed to three outcome events as protective or risk factors of disease progression in the early stage of liver cirrhosis (**Supplementary Table S2**).

**Figure 2:**
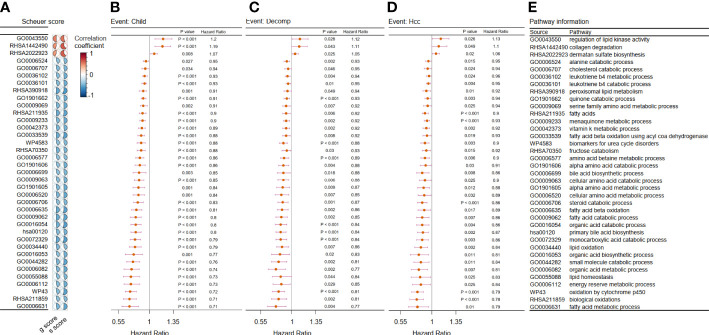
Filtration of metabolic pathways related to disease progression. **(A–D)** We showed only the 37 overlapping outcome-related pathways in Cohort 1 and Cohort 2. **(A)** Heatmap showing the correlation coefficient between the chosen metabolic pathways and two kinds of liver biopsy in Cohort 1. Forest plot of univariate Cox analysis for the chosen metabolic pathways related with **(B)** evaluation of progression of Child–Pugh class (child), **(C)** hepatic decompensation (decomp), and **(D)** hepatocellular cancerization (hcc) in Cohort 2 (p < 0.05). **(E)** Related metabolic pathway in panels **(A–D)**.

After the integration of all the metabolic pathways associated with disease progression in the two cohorts, 37 common metabolic pathways were identified. Of these, three pathways were identified as risk factors, while the remaining pathways were protective (**Figure 2**).

### 3.3 Construction of models related to viral hepatitis disease progression risk

**Figure 3:**
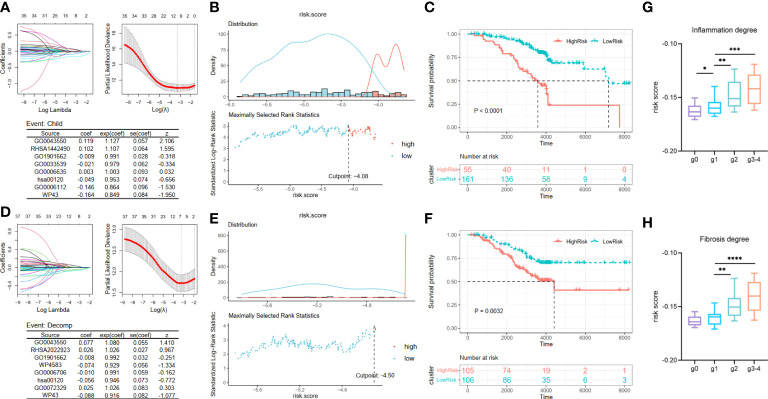
Construction and validation of liver function deterioration progression risk-related models. For the two events, **(A, D)** LASSO regression models were visualized. coef, regression coefficient; exp(coef), exponent coefficient (hazard ratio); se(coef), standard error of the coefficient; z, Wald statistic. **(B, E)** According to the cut point derived from maximally selected rank statistics, the patients were separated into two risk subgroups. **(C, F)** Kaplan–Meier plots of survival curve between high-risk and low-risk groups in two models. **(G, H)** Box plot showing a difference in Child–Pugh class progression related-risk score among the five grades in two evaluation approaches. LASSO, least absolute shrinkage and selection operator. ****means p < 0.0001,*** means p < 0.001, ** means p < 0.01, and * means p < 0.05.

Considering that an excessively large number of pathways may cause a drop in the value of the clinical application, we employed the LASSO Cox regression analysis to narrow the range of metabolic pathways and constructed risk models from two aspects: deterioration of liver function and carcinogenesis development. Two risk assessment models of liver function deterioration progression were built according to two events in Cohort 2: child and decomp. First, after LASSO Cox regression analysis, eight optimal variables of Child–Pugh class progression were extracted from 37 metabolic pathways mentioned above to construct the signature (**Figure 3A**). Then, the corresponding coefficients obtained from the multivariate Cox regression model and then ssGSEA scores of the optimal eight pathways were combined to obtain a Child–Pugh class progression risk score for each patient: score1 = 0.119 * GO0043550 + 0.102 * RHSA1442490 − 0.009 * GO1901662 − 0.021 * GO0033539 + 0.003 * GO0006635 − 0.049 * hsa00120 − 0.146 * GO0006112 − 0.164 * WP43. Based on the optimal cutoff value of the risk score, the patients could be stratified into high- and low-risk groups (**Figure 3B**). Then, a Kaplan–Meier curve was utilized to show that patients in the high-risk subgroup have significantly worse prognoses than their low-risk counterparts (**Figure 3C**, p < 0.0001). Similarly, we attained eight optimal hepatic decompensation-related pathways (**Figure 3D**) and corresponding risk score: score2 = 0.077 * GO0043550 + 0.026 * RHSA2022923 − 0.008 * GO1901662 − 0.074 * WP4583 − 0.010 * GO0006706 − 0.056 * hsa00120 + 0.025 * GO0072329 − 0.088 * WP43. However, if we took the optimal value determined with the maximally selected rank method as the cut point of the two groups, the sample size of the high-risk group might be so small that it interfered with later survival analysis (**Figure 3E**). Thus, the high- and low-risk groups were divided in accordance with the median value of the risk score. Then, the striking difference between these two subgroups is also shown in **Figure 3F**. Moreover, we noted the relationship between the scale of inflammation and fibrosis and the risk score of liver function deterioration. Considering the small number of g3 and g4 grades, we regard the two grades as one group. The results suggest that patients with a higher Scheuer score for their liver biopsy have a greater risk score of Child–Pugh class progression (**Figures 3G, H**).

**Figure 4:**
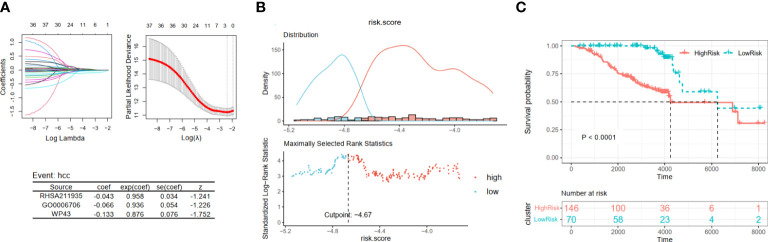
Construction and validation of viral hepatitis cancerization risk-related models. **(A)** The LASSO regression model is visualized. **(B)** According to the cut point with maximally selected rank statistics, the patients were split into high-risk and low-risk groups. **(C)** Kaplan–Meier plots of survival curve between two subgroups. LASSO, least absolute shrinkage and selection operator.

Then, by taking the occurrence of liver cancer as the outcome of each patient in Cohort 2, we established the HCC development risk assessment model. Their key pathways were also identified from the above 37 pathways employing LASSO Cox regression analysis to build the HCC development risk assessment model: score3 = −0.043 * RHSA211935 − 0.066 * GO0006706 − 0.133 * WP43 (**Figure 4A**). The patients were divided into two subgroups based on the optimal cut point, and the difference between the two subgroups was significant, as demonstrated by the results of the analysis (**Figures 4B, C**).

### 3.4 Construction of a viral hepatitis prognostic model based on prognosis-related metabolic pathways

**Figure 5:**
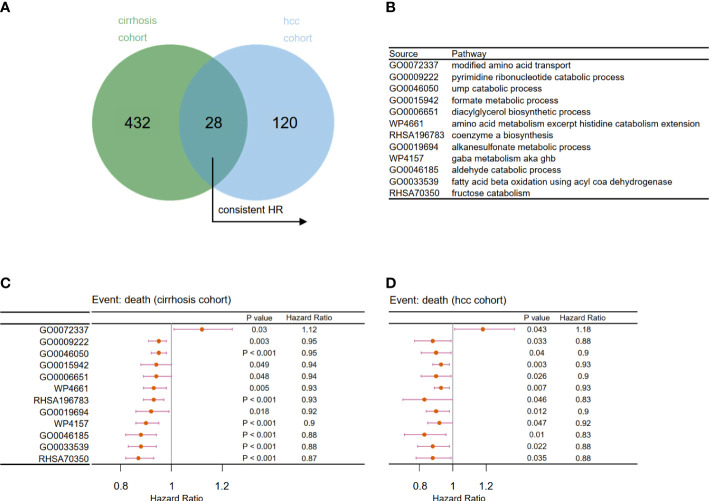
Filtration of prognosis-related metabolic pathways. **(A)** Venn diagram to identify 28 overlapping prognosis related-metabolic pathways. **(B)** Related metabolic pathways in panels **C** and **D**. Forest plot of univariate Cox analysis for the chosen prognosis-related metabolic pathways in **(C)** Cohort 2 and **(D)** Cohort 3 (p < 0.05).

Next, we examined the effects of metabolic pathways on the survival and prognosis of viral hepatitis patients from the perspectives of the cancer-free and cancerous stages. The Cox regression model was used to assess the effects of each metabolic pathway on the prognosis of patients in Cohort 2 and Cohort 3. Finally, 28 common metabolic pathways (**Figures 5A, B**) were identified, of which 12 pathways have a consistent trend of hazard ratio (**Figures 5C, D**). This indicates that these 12 pathways contributed greatly to determining the prognosis of viral hepatitis patients before and after liver cancer development. To establish prognostic risk models, we will examine these 12 pathways for key pathways.

**Figure 6:**
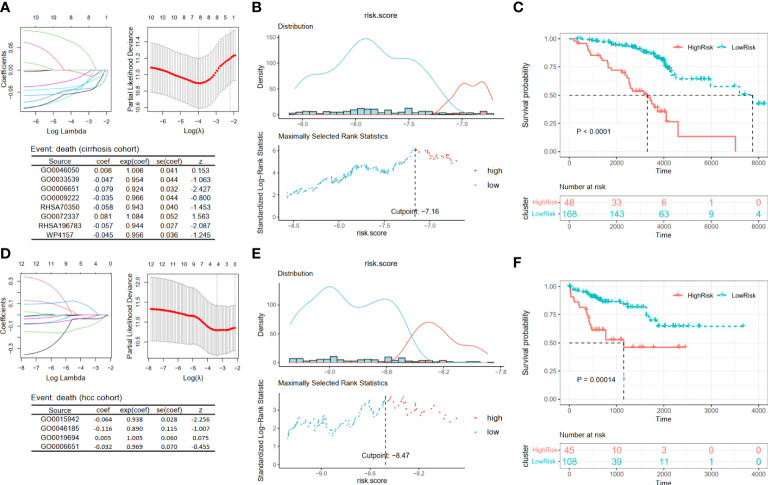
Construction and validation prognosis-related models. **(A, D)** The LASSO regression models were visualized. **(B, E)** According to the cut point obtained from maximally selected rank statistics, the patients were divided into two risk groups. **(C, F)** Kaplan–Meier plots of survival curve between high-risk and low-risk groups in two models. LASSO, least absolute shrinkage and selection operator.

For patients in Cohort 2, we selected eight pathways by LASSO Cox regression analysis and constructed a prognostic model for patients before the occurrence of liver cancer: score4 = 0.006 * GO0046050 − 0.047 * GO0033539 − 0.079 * GO0006651 − 0.035 * GO0009222 − 0.058 * RHSA70350 + 0.081 * GO0072337 − 0.057 * RHSA196783 − 0.045 * WP4157 (**Figure 6A**). There was a significant difference between the high- and low-risk groups, which were separated by an appropriate value (**Figures 6B, C**, p < 0.0001). Similarly, based on the survival data of liver cancer patients in Cohort 3, we obtained their prognostic model: score5 = −0.064 * GO0015942 − 0.116 * GO0046185 + 0.005 * GO0019694 − 0.032 * GO0006651 (**Figure 6D**). Then, we obtained two groups and also found that patients in the low-risk group had a better prognosis (**Figures 6E, F**, p = 0.00014).

### 3.5 Metabolic disorders mediate the imbalance of immune homeostasis in hepatitis

**Figure 7:**
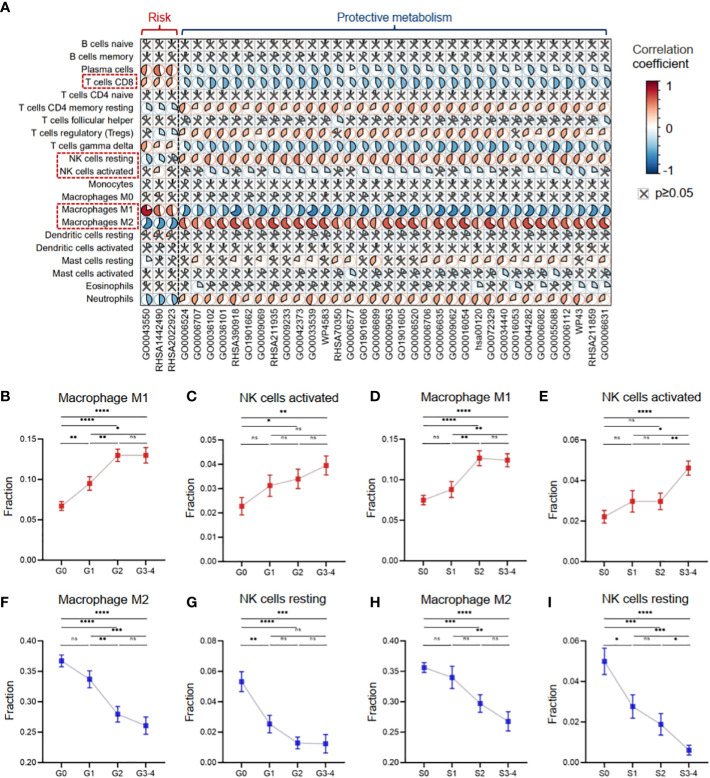
Metabolic disorders contributed to the immune imbalance in hepatitis. **(A)** Heatmap showing Pearson’s correlation coefficients between the activities of metabolic pathways and the immune infiltration fractions. **(B)** The infiltration fraction of M1 macrophages in groups with different degrees of inflammation. **(C)** The infiltration fraction of activated NK cells in groups with different degrees of inflammation. **(D)** The infiltration fraction of M1 macrophages in groups with different degrees of fibrosis. **(E)** The infiltration fraction of activated NK cells in groups with different degrees of fibrosis. **(F)** The infiltration fraction of M2 macrophages in groups with different degrees of inflammation. **(G)** The infiltration fraction of resting NK cells in groups with different degrees of inflammation. **(H)** The infiltration fraction of M2 macrophages in groups with different degrees of fibrosis. **(I)** The infiltration fraction of resting NK cells in groups with different degrees of fibrosis. Data in panels **B**–**I** are mean ± SEM. LASSO, least absolute shrinkage and selection operator. ****means p < 0.0001,*** means p < 0.001, ** means p < 0.01, and * means p < 0.05.

Furthermore, we investigated the role of the 37 critical metabolic pathways in disease progression from the immunological perspective. In Cohort 1 with clinical information on inflammatory activity and fibrosis degree, we evaluated 22 types of immune cell infiltration fractions by CIBERSORTx, and then the correlation between the activities of the 37 critical metabolic pathways and the immune infiltration fractions was calculated (**Figure 7A**). The results demonstrated that three risk metabolic pathways showed obvious immune-enhancing and pro-inflammatory effects: their metabolic activity was positively associated with plasma cells, gamma delta T cells, M1 macrophages, CD8+ T cells, and activated NK cells but negatively associated with resting memory CD4+ T cells, Treg cells, resting NK cells, and M2 macrophages. In contrast, the remaining protective metabolic pathways had immunosuppressive and anti-inflammatory effects (**Figure 7A**).

Our results revealed distinct effects of metabolic pathways on different subsets of macrophages and NK cells. Specifically, liver tissues with high inflammation and fibrosis levels had increased infiltration of pro-inflammatory M1 macrophages and activated NK cells, which were associated with risk metabolic pathways (**Figures 7B–E**). In contrast, liver tissues with high inflammation and fibrosis levels had decreased infiltration of anti-inflammatory M2 macrophages and resting NK cells, which were associated with protective metabolic pathways (**Figures 7F–I**).

### 3.6 Intrahepatic single-cell immune map reveals the metabolic perturbations of macrophage, NK cell, and CD8+ T-cell clusters

To further understand the effects of intrahepatic metabolic dysregulation on the immune cell subtypes, we utilized the scRNA-seq profiles of immune cells in liver tissues. In total, 19 cases were enrolled, including 15 HBV-infected cases (HBV) and four healthy control cases (HC). After quality control and batch correction, 98,074 cells were retained for analysis. There were 11 cell types identified according to the expression level of the canonical cell markers (**Supplementary Figures 1A, B**).

Next, we evaluated the degree of metabolic imbalances in various cell types in HBV infection. The expression of 711 metabolic enzyme genes (see Materials and Methods) in each cell was included in the analysis. Machine-learning framework (random forest algorithm) was used to determine the responsiveness of each cell subset to biological perturbations caused by HBV infection. The results indicated that macrophages were the most responsive cell type to biological perturbations, with an AUC value of 0.832. NK cells had moderate responsiveness, with an AUC of 0.611 (**Supplementary Figures 1C, D**).

**Figure 8:**
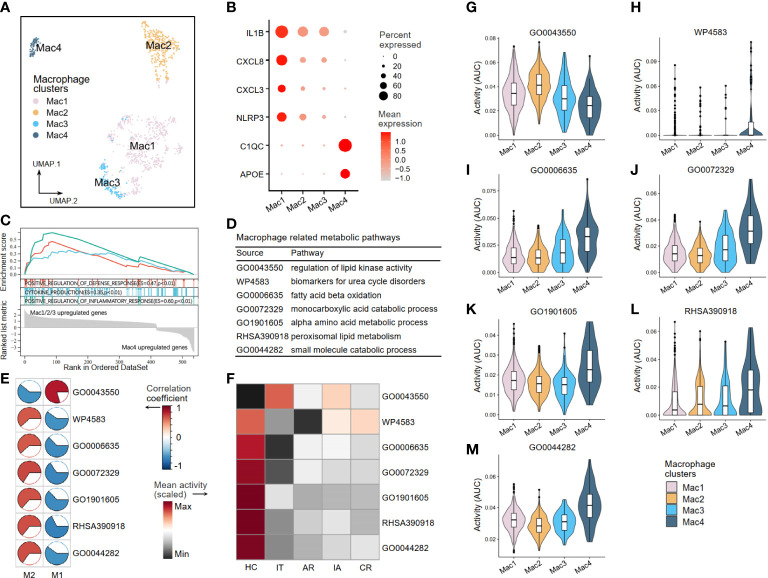
The metabolic perturbations of macrophage clusters in hepatitis. **(A)** UMAP visualization of the macrophage clusters. **(B)** Dot plot showing the expression levels of given genes in each cluster. **(C)** GSEA of the significant DEGs between Mac1–3 and Mac4. An enrichment score (ES) > 0 indicated that the pathway is upregulated in Mac1–3, while an ES < 0 indicated that the pathway is upregulated in Mac4. **(D)** Macrophage-related metabolic pathways and corresponding database sources. **(E)** Heatmap showing Pearson’s correlation coefficients between the activities of metabolic pathways and the Macrophage infiltration fractions. **(F)** Heatmap showing the scaled mean activities of metabolic pathways in each group. HC, healthy control; IT, immune tolerant phase; AR, acute recovery phase; IA, immune active phase; CR, chronic resolved phase. **(G–M)** The activities of metabolic pathways in each macrophage cluster. UMAP, Uniform Manifold Approximation and Projection; GSEA, gene set enrichment analysis; DEGs, differentially expressed genes.

A second round of clustering was conducted to better understand the metabolic perturbations of macrophages and NK cells. The AUCell analysis was used to evaluate pathway activity based on the given 37 critical metabolic pathways (see **Figure 2**) in the scRNA-seq dataset. Four macrophage clusters (Mac1–4) were generated by re-clustering (**Figure 8A**). The Mac1–3 clusters expressing high levels of inflammatory molecules (IL1β, CXCL8, CXCL3, and NLRP3) were annotated as pro-inflammatory clusters. The Mac4 clusters expressing low levels of inflammatory molecules but high levels of C1QC and APOE were considered resting or anti-inflammatory (**Figure 8B**). Other DEGs are shown in **Supplementary Table S3**. The GSEA was applied to identify functional differences in immune pathways based on the DEGs between Mac1–3 and Mac4. The results suggested that Mac1–3 had enhanced defense response, cytokine production, and inflammatory response (**Figure 8C**). Next, we verified seven macrophage-related metabolic pathways in scRNA-seq (**Figure 8D**). In addition to the regulation of lipid kinase activity pathway (GO0043550), which was a pro-inflammatory metabolic pathway, the other six were anti-inflammatory pathways, including urea cycle disorders (WP4583), fatty acid beta-oxidation (GO0006635), monocarboxylic acid metabolism (GO0072329), alpha amino acid metabolism (GO1901605), peroxisomal lipid metabolism (RHSA390918), and small molecule metabolism (GO0044282) (**Figure 8E**). We found that the activity of the GO0043550 pathway was higher than that in the HC group at all stages (acute recovery (AR), immune tolerant (IT), immune active (IA), and chronic resolved (CR)) of HBV infection. In comparison, the other six anti-inflammatory pathways had decreased activity in HBV groups (**Figure 8F**). As expected, the activity level of the pro-inflammatory pathway GO0043550 was high in pro-inflammatory clusters Mac1–3 but low in resting clusters Mac4 (**Figure 8G**). In contrast, the other six anti-inflammatory pathways had high activities in the resting cluster Mac4 (**Figures 8H–M**).

**Figure 9:**
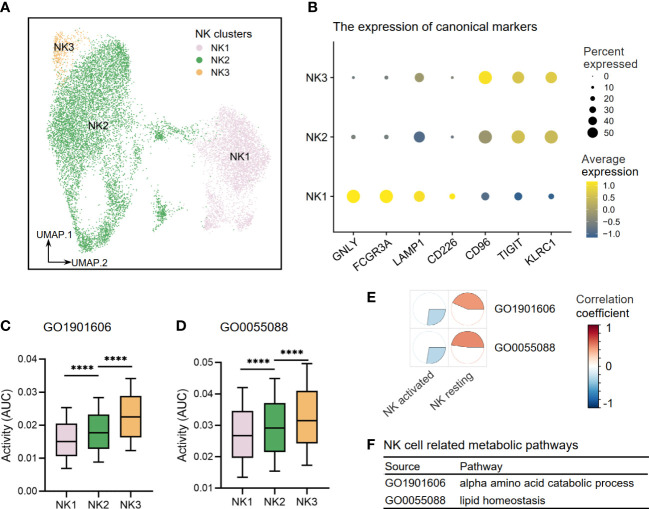
The metabolic perturbations of NK clusters in hepatitis. **(A)** UMAP visualization of the NK clusters. **(B)** Dot plot showing the expression levels of given genes in each cluster. **(C, D)** The activities of metabolic pathways in each NK cluster. **(E)** Heatmap showing Pearson’s correlation coefficients between the activities of metabolic pathways and the NK infiltration fractions. **(F)** NK-related metabolic pathways and corresponding database sources. UMAP, Uniform Manifold Approximation and Projection. ****means p < 0.0001.

Through re-clustering, three clusters of NK cells (NK1–3) were annotated (**Figure 9A**). NK1 subset expressed high levels of CD226 (DNAX Accessory Molecule-1), a costimulatory molecule that indicates an activation state of NK cells with cytotoxic effector and cytokine production functions. NK1 also expressed high levels of GNLY (Granulysin), FCGR3A (Fc Gamma Receptor IIIa), and LAMP1 (lysosomal-associated membrane protein 1), which suggested strong cell-killing and degranulation abilities. NK2–3 subsets expressed high levels of inhibitory molecules CD96 (Tactile), TIGIT (T-cell immunoglobulin and ITIM domain), and KLRC1 (Killer Cell Lectin Like Receptor C1), indicating that they were inactive or resting (**Figure 9B**). Other DEGs are shown in **Supplementary Table S3**. Furthermore, we investigated the activities of NK cell-related metabolic pathways in three clusters of NK cells and found that the activities of the alpha-amino acid metabolism pathway (GO1901605) and lipid homeostasis pathway (GO0055088) were upregulated in resting clusters of NK2–3 (**Figures 9C, D**). Interestingly, our correlation analysis results in Cohort 1 supported these findings by demonstrating that activated NK cells are more closely associated with disease risk-related metabolic processes than resting NK subsets, as illustrated in **Figures 9E, F**.

**Figure 10:**
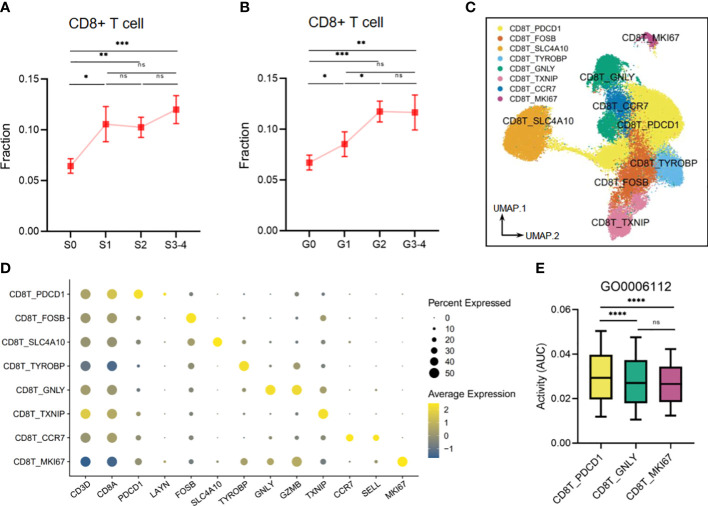
The metabolic perturbations of CD8T clusters in hepatitis. **(A)** The infiltration fraction of CD8+ T in groups with different degrees of fibrosis. **(B)** The infiltration fraction of CD8+ T in groups with different degrees of inflammation. **(C)** UMAP visualization of the CD8T clusters. **(D)** Dot plot showing the expression levels of given genes in each cluster. **(E)** The activities of the metabolic pathway in several CD8T clusters. Data in panels **A** and **B** are mean ± SEM. UMAP, Uniform Manifold Approximation and Projection. ****means p < 0.0001,*** means p < 0.001, ** means p < 0.01, and * means p < 0.05.

Our findings also demonstrated that liver tissues with high levels of inflammation and fibrosis exhibit an elevated infiltration of CD8+ T cells, which are linked to risk metabolic pathways (**Figures 10A, B**). Upon re-clustering, we observed various CD8+ T-cell clusters (**Figure 10C**), which were identified through the expression of their characteristic markers, CD3D and CD8A. One of these clusters, CD8T_PDCD1, expressed high levels of PDCD1 and LAYN, which are typical markers of exhausted T cells. Additionally, FOSB (G0/G1 switch regulatory protein 3), SLC4A10 (Solute carrier family 4 member 10), TYROBP (Transmembrane immune signaling adaptor TYROBP), and TXNIP (Thioredoxin-interacting protein) were utilized to annotate the corresponding CD8+ T-cell clusters. Another cluster, CD8T_GNLY, expressed high levels of the activation marker, granzymes (GZMB), and granulysin (GNLY), representing a high cytotoxicity of this CD8+ T subset. The CD8T_CCR7 subset exhibited high expression of CCR7 and SELL(CD62L), indicating a naive T subset. Finally, the CD8+ T-cell cluster with high expression of MKI67 suggested an active proliferation future (**Figure 10D**). Other DEGs are shown in **Supplementary Table S3**. Next, we analyzed the activity of the disease-related metabolic pathway mentioned earlier (**Figures 2E**, **7A**) in the CD8+ T-cell clusters. We observed that the activity of a protective pathway, the energy reserve metabolic process (GO0006112), was upregulated in the CD8T_PDCD1 subset compared to the CD8T_GNLY and CD8T_MKI67 subsets (**Figure 10E**). This may indicate that CD8+ T cells can maintain the balance of killer-proliferative cluster and exhausted cluster by the energy reserve metabolic homeostasis.

## 4 Discussion

In this study, we used ssGSEA scores of metabolic pathways in viral hepatitis patients to identify the most active pathways and created risk assessment models for disease progression and prognosis. These models were validated through survival analysis and exhibited excellent capacities for risk prediction. Additionally, we investigated the potential mechanism by analyzing immune infiltration using single-cell transcriptomics data. Our findings suggested that the dysfunction of liver metabolism can cause immune dysregulation and highlighted the crucial role of metabolic dysfunction of NK, macrophage, and CD8+ T subsets in disease development.

To ensure that our disease progression risk models have a high predictive value, we used liver histopathology, function deterioration, and carcinogenesis as evaluation indicators and screened for metabolic pathways related to the risk of disease progression. Moreover, to better align our findings with the demands of a modern personalized treatment model, we narrowed the scope of these pathways, attempted to identify key metabolic pathways, and constructed disease progression risk models based on these pathways. Our results showed that there are three common key metabolic pathways in the two risk models of liver function deterioration progression, in which regulation of lipid kinase activity is a risk pathway, while quinone catabolic process and primary bile acid biosynthesis are protective. This result indicated that they are vital in the process of liver function deterioration. An endosomal protein has been shown to trigger an autophagy response during viral infections by increasing lipid kinase activity, which may also contribute to the progression of viral hepatitis (Dong et al., 2021). Redox reactions triggered by quinone can lead to damage to hepatocytes. To counteract this, key enzymes such as NAD(P)H quinone dehydrogenase 1 (NQO1) decompose these quinone compounds to protect hepatocytes (Dinkova-Kostova and Talalay, 2000; Kudoh et al., 2014). The liver plays a crucial role in both bile acid biosynthesis and steroid catabolism. According to the model, the primary bile acid biosynthesis pathway appears to be a good predictor of liver function, while the steroid catabolic process is more closely linked to late-stage liver deterioration and the risk of liver cancer. The liver is the major apparatus for breaking down poisonous substances, and cytochrome p450 plays a crucial role in this process. Disruptions in this process can lead to liver fibrosis, inflammation, and eventually liver cancer due to toxicant buildup (Backman et al., 2016; Gao et al., 2020). In line with this, our results showed that oxidation by cytochrome p450 is a key indicator for predicting risk during all phases of viral hepatitis disease progression. Furthermore, to make our models more comprehensive and individualized, we preliminarily screened the prognosis related-metabolic pathways through survival data of non-cancer and cancer viral hepatitis and identified key pathways from these two to establish two prognostic models. Although the key metabolic pathways were different between the two models, there was a common metabolic pathway between them: the diacylglycerol biosynthetic process. Diacylglycerol is an important component of cellular membranes and glycerol lipids and has a crucial impact on various metabolic programs and signaling pathways (Eichmann and Lass, 2015). All these suggest that diacylglycerol biosynthesis is an excellent predictor of both non-cancer and cancer viral hepatitis, likely due to its involvement in a variety of prognosis-related metabolic processes.

To investigate the contribution of immune cells in metabolic processes, we performed an analysis of immune cell infiltration. Our analysis revealed that several immune cells, such as macrophages, NK cells, and CD8+ T cells, have a significant impact on metabolic pathways. By combining the results from the pathological samples, we identified two distinct subsets of macrophages (Shapouri-Moghaddam et al., 2018), classically activated or inflammatory (M1) macrophages, which have a high correlation with increased disease risk, while alternatively activated or anti-inflammatory (M2) macrophages were found to offer protection. Moreover, we observed similar characteristics in two subsets of NK cells. CD8+ T cells showed a high correlation with increased disease risk. Furthermore, we explored the disease metabolic disorder on these immune subsets in different states, including classical cell types and some re-clustering subsets.

In our study, we identified activated macrophage clusters, Mac1–3, based on their levels of anti-inflammatory or pro-inflammatory cytokines, such as IL1β, NLRP3, and pro-inflammatory CXC chemokines, as previously reported (Ley, 2008; Renaudin et al., 2020). APOE is an anti-inflammatory molecule that can be produced by mononuclear cells in response to inflammatory cytokines such as IL1β (Ali et al., 2005; Braesch-Andersen et al., 2013). Therefore, the Mac4 subset, which had high levels of APOE and other anti-inflammatory cytokines, was classified as a resting macrophage cluster. This classification was consistent with the enrichment analysis of inflammatory functions, which showed that the Mac4 subset had lower levels of inflammatory pathways than the Mac1–3 subsets. By scRNA-seq analysis of macrophage-related metabolic pathways, we identified increased activity of the lipid kinase pathway in activated macrophage subsets, such as M1 macrophages and Mac1–3 clusters. During each stage of HBV infection, the activity of this pathway was found to be higher compared to that of the healthy control group (Zhang et al., 2023). It has previously been demonstrated that lipid kinases regulate macrophage immune responses and participate in tumor progression and metastasis in pancreatic ductal adenocarcinoma (Kaneda et al., 2016). Unlike the lipid kinase pathway, which is active in inflammatory macrophage subsets (Mac1–3), the peroxisomal lipid metabolism pathway is highly active in resting macrophage subsets (M2 macrophages and Mac4). Peroxisomes are organelles that participate in various types of lipid metabolism and have important roles in viral hepatitis and fatty liver disease (Dharancy et al., 2005; Reddy and Rao, 2006; Kim et al., 2007). Our findings added to our understanding of the potential mechanisms by indicating that resting macrophage subsets may be responsible for the protective effects of peroxisomal lipid metabolism.

To estimate the activity states of the three NK cell clusters, we evaluated the expression of canonical markers related to their activation or inactive state and cell-killing and degranulation functions. NK cells can be activated by CD226, whereas CD96 and TIGIT binding to CD226 ligands can play an inhibitory effect and balance out the cytotoxicity of NK cells that are activated by CD226 (Martinet and Smyth, 2015). GNLY, FCGR3A, and LAMP1 may effectively depict active states as NK cell signatures for cytokine generation and cytotoxicity, while KLRC1 serves a crucial inhibitory role in controlling NK cells’ activation and effector activities (Braud et al., 1998; Alter et al., 2004; Mathewson et al., 2021). The results of our study revealed that resting state NK cell subsets exhibit high levels of activity in both alpha amino acid catabolism and lipid homeostasis, as determined by both classical classification and re-clustering analysis. It was implied that there may be a relationship between NK cells and the two metabolic pathways that can potentially reduce the risk of viral hepatitis progression. This connection may be through the effect of these pathways on the resting state of NK cells. The study by X. Fang et al. provided evidence that NK cells not only possess strong cell-killing abilities but also are able to maintain homeostasis in lipid metabolism in trophoblasts by increasing the expression of apolipoprotein APOD, which helps preserve a normal state in these cells (Fang et al., 2022). There may be a similar lipid homeostasis-related process that would occur in resting NK cells for patients with viral hepatitis to slow down the spread of liver cell damage.

Upon assessing the metabolic imbalances in various immune cell types during HBV infection, our results showed that the response of CD8 T cells was weaker in comparison to that of macrophages and NK cells. Nevertheless, it should be acknowledged that CD8 T cells still play a vital role in the immune response in the development of viral hepatitis. The prevailing consensus holds that CD8 T cells play an indispensable role in the clearance of hepatitis virus infections (Guidotti and Chisari, 2006) while simultaneously producing certain antiviral cytokines (Guidotti, 2002). These processes are believed to be advantageous in impeding the progression of viral hepatitis. Nonetheless, it is noteworthy that the elimination of viruses by CD8 T cells is often accompanied by hepatic cell destruction, which not only directly results in liver impairment (Maini et al., 2000) but also induces liver fibrosis due to aberrant hepatic cell regeneration and repair of the liver, culminating in the ultimate progression to cirrhosis and hepatocellular carcinoma (Nakamoto et al., 1998; Sitia et al., 2012). Our findings also indicate that infiltration of CD8+ T cells may exacerbate inflammation and fibrosis in liver cells, demonstrating the damaging effect of CD8+ T cells on the liver. Furthermore, we conducted a re-clustering of intrahepatic CD8+ T cells and classified each cluster based on the expression of several essential functional genes. Compared to activated phenotype clusters, such as CD8T_GNLY and CD8T_MKI67, which demonstrate cytotoxic and proliferative characteristics, exhausted T cells (CD8T_PDCD1) exhibit a higher energy reserve metabolic activity. The crucial role of energy for proper immune cell function is evident, especially in chronic inflammatory states where immune cells require a higher priority for energy demands compared to other body parts (Straub et al., 2010). Our results suggest that maintaining energy reserve metabolic homeostasis may alleviate CD8+ T cell-mediated liver damage by balancing the killer-proliferative and exhausted clusters, thus slowing down disease progression.

This study has several limitations. Although the results of our models are confirmed by the K-M curve, experimental verification is lacking. Additionally, our prediction models have the potential to provide insights for clinical decision-making, but it is important to note that further clinical testing and the development of guidelines may be necessary for the future to ensure the practicality and effectiveness of our prediction model.

## 5 Conclusion

In conclusion, our study offered a unique perspective on the development and prognosis of viral hepatitis by utilizing the biological process of metabolism. Our findings resulted in the creation of a novel risk assessment tool, providing a valuable resource for clinicians in making informed decisions. Furthermore, our work provides new insights into the relationship between viral hepatitis and immunometabolism, enriching our understanding of the underlying mechanisms involved in this disease.

## Data availability statement

The datasets presented in this study can be found in online repositories. The names of the repository/repositories and accession number(s) can be found in the article/**Supplementary Material**.

## Author contributions

XYL and BZ conceived and directed the completion of the study. MZ and YL performed data analysis. The manuscript was drafted by MZ, YL, YZ, and MS and was revised by XL, ZZ, and JH. All authors contributed to the article and approved the submitted version.

## Supplementary material

Supplementary Figure 1 Single cell immune landscape of liver tissues infected with HBV. **(A)** UMAP visualization of the immune cell subtypes in liver tissues. **(B)** Dot plot exhibiting the expression levels of canonical markers in each cell type. **(C)** UMAP plot annotated with cell types and AUC values. **(D)** The AUC value of each immune cell type.

Supplementary Table 1 Names and corresponding correlation analysis results of 220 metabolic pathways associated with both liver inflammation and fibrosis in Cohort1.

Supplementary Table 2 Names and corresponding risk assessment results of 96 metabolic pathways associated with risk of liver dysfunction and hepatocellular carcinogenesis in Cohort 2.

Supplementary Table 3 Differential Expressed Genes of various macrophage, NK, and CD8+T cell clusters.
