# Supplementary figures and images for: Development and investigation of metabolism-associated risk assessment models for patients with viral hepatitis
Source: Front Cell Infect Microbiol. 2023 Mar 29;13:1165647. doi: 10.3389/fcimb.2023.1165647 (PMC10095836; doi:10.3389/fcimb.2023.1165647)

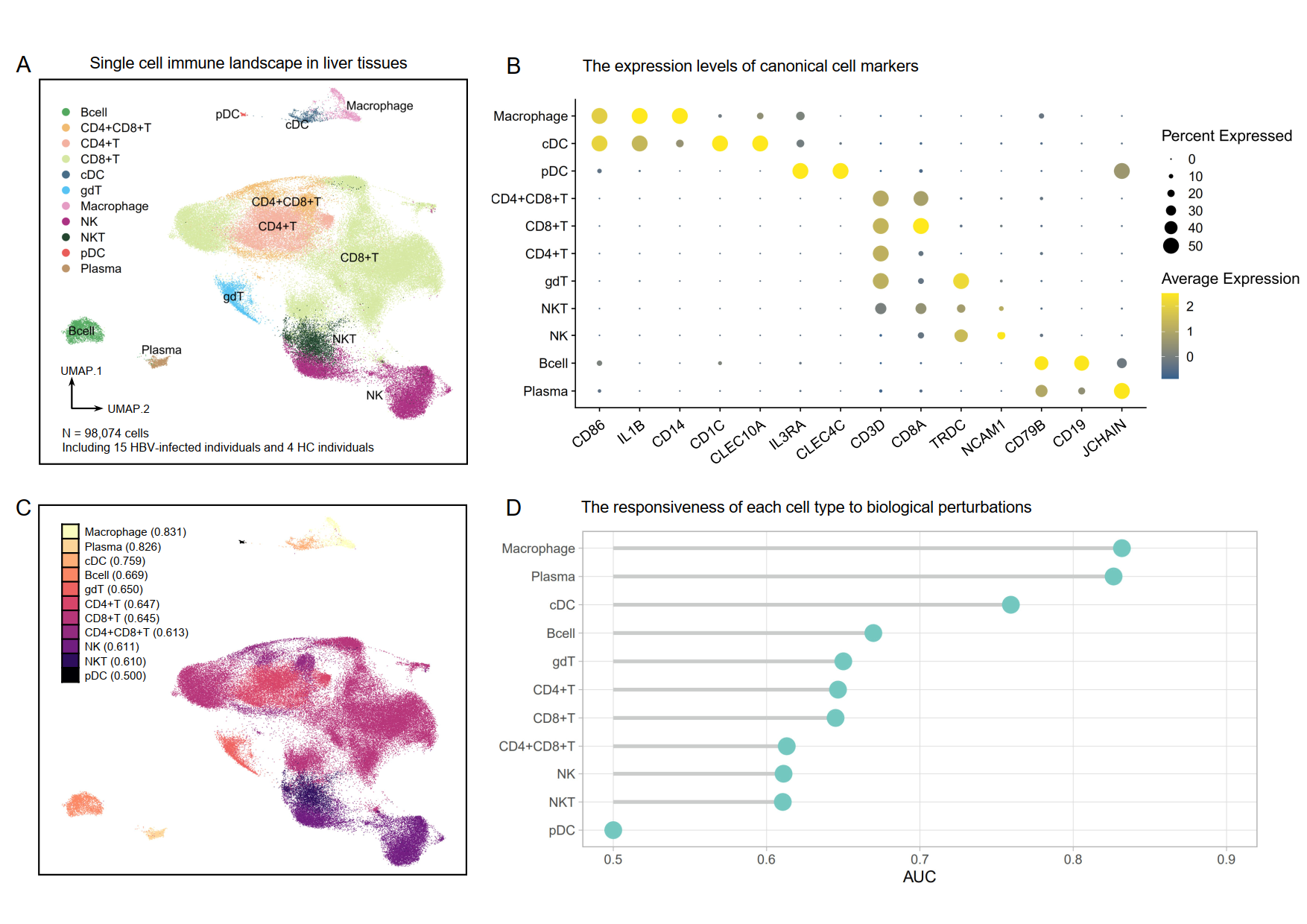

Supplement: Supplementary Figure 1 — Single cell immune landscape of liver tissues infected with HBV. (A) UMAP visualization of the immune cell subtypes in liver tissues. (B) Dot plot exhibiting the expression levels of canonical markers in each cell type. (C) UMAP plot annotated with cell types and AUC values. (D) The AUC value of each immune cell type. [file Image_1.tif]
