# Supplementary material for: Development and investigation of metabolism-associated risk assessment models for patients with viral hepatitis
Source: Front Cell Infect Microbiol. 2023 Mar 29;13:1165647. doi: 10.3389/fcimb.2023.1165647 (PMC10095836; doi:10.3389/fcimb.2023.1165647)
Supplement: Supplementary file 5 [file DataSheet_1.pdf]

Supplementary Material

Development and Investigation of Metabolism Associated Risk Assessment Models for Patients with Viral Hepatitis

Mingjiu Zhao[1†], Yu Lei[2†], Yanyan Zhou[3], Mingan Sun[4], Xia Li[1], Zhiguang Zhou[1],Jiaqi Huang[1,5], Xinyu Li[1*], Bin Zhao[1,6*]

[1]National Clinical Research Center for Metabolic Diseases, Metabolic Syndrome Research Center, Key Laboratory of Diabetes Immunology, Ministry of Education, and Department of Metabolism and Endocrinology, The Second Xiangya Hospital of Central South University, Changsha, Hunan, China.
[2]Department of Dermatology, Hunan Key Laboratory of Medical Epigenomics, The Second Xiangya Hospital of Central South University, Changsha, Hunan, China.
[3]Department of Critical Care Medicine, The Second Xiangya Hospital of Central South University, Changsha, Hunan, China.
[4]College of Veterinary Medicine, Yangzhou University, Yangzhou, Jiangsu, China.
[5]Xiangya School of Public Health, Central South University, Changsha, China.
[6]Furong Laboratory, Central South University, Changsha, China.

† Mingjiu Zhao and Yu Lei have contributed equally to this work and share first authorship
* Bin Zhao and Xinyu Li jointly supervised this work
* **Correspondence**:
Bin Zhao
binzhao@csu.edu.cn bin.zhao@live.com
Xinyu Li
2204170324@csu.edu.cn

Supplementary figure 1 | Single cell immune landscape of liver tissues infected with HBV. (**A**) UMAP visualization of the immune cell subtypes in liver tissues. (**B**) Dot plot exhibiting the expression levels of canonical markers in each cell type. (**C**)

UMAP plot annotated with cell types and AUC values. (**D**) The AUC value of each immune cell type.

Supplementary Table S1 | Names and corresponding correlation analysis results of 220 metabolic pathways associated with both liver inflammation and fibrosis in Cohort1.

Supplementary Table S2 | Names and corresponding risk assessment results of 96 metabolic pathways associated with risk of liver dysfunction and hepatocellular carcinogenesis in Cohort2.

Supplementary Table S3 | Differential Expressed Genes of various macrophage, NK, and CD8+T cell clusters.
